# Post-influenza bacterial infection: mechanisms of pathogenesis and advances in therapeutic strategies

**DOI:** 10.3389/fmicb.2025.1673643

**Published:** 2025-10-08

**Authors:** Biao Lei, Shun Wang, Linzhong Yu, Qinhai Ma

**Affiliations:** ^1^Southern Medical University Hospital of Integrated Traditional Chinese and Western Medicine, Southern Medical University, Guangzhou, Guangdong, China; ^2^School of Traditional Chinese Medicine, Shanghai University of Traditional Chinese Medicine, Shanghai, China; ^3^Third Level Research Laboratory of State Administration of Traditional Chinese Medicine, School of Traditional Chinese Medicine, Southern Medical University, Guangzhou, Guangdong, China; ^4^Guangdong Provincial Key Laboratory of Chinese Medicine Pharmaceutics, School of Traditional Chinese Medicine, Southern Medical University, Guangzhou, Guangdong, China; ^5^State Key Laboratory of Respiratory Disease, National Clinical Research Center for Respiratory Disease, Guangzhou Institute of Respiratory Health, The First Affiliated Hospital of Guangzhou Medical University, Guangzhou Medical University, Guangzhou, Guangdong, China

**Keywords:** post-influenza bacterial infection, resident immune cells, respiratory barrier, therapeutic strategies, overactivated inflammatory response

## Abstract

Patients coinfected with influenza virus (IFV) and bacteria face significantly elevated risks of critical illness and mortality. This vulnerability stems primarily from IFV-induced immunosuppression and disruption of respiratory barrier integrity. Specifically, prior IFV infection compromises the airway epithelium and impairs immune cell function, creating a permissive environment for secondary bacterial infections that drive severe disease progression. Within the lung, resident immune cells are crucial for pathogen surveillance, antibacterial defense, and homeostasis maintenance. However, recruited neutrophils and macrophages paradoxically become key drivers of detrimental immunopathology during coinfection. The literatures involved in influenza bacterial infection, influenza bacterial superinfection, post-influenza bacterial infection and secondary bacterial infection, were included. In this review, we summarize the literatures about epidemiology, treatment options and two pivotal mechanisms: The primary mechanisms of IFV-mediated susceptibility to bacterial infection, focusing on epithelial barrier damage and immune cell dysfunction; the central roles of specific immune cells (notably neutrophils and macrophages) and their effector pathways in fueling hyperinflammatory responses that cause severe immunopathology. A comprehensive understanding of the interactions between the pathogens and the host will assist in the development of therapeutic modalities for the prevention and treatment of post-influenza bacterial infection.

## 1 Introduction

Lower respiratory infections ranked as the seventh-highest global cause of death in 2021, resulting in more than 2 million deaths in the world (Naghavi et al., 2024). Influenza virus (IFV) infection is one of the most common factors leading to lower respiratory infections (Troeger et al., 2019; Li et al., 2021). Patients with IFV infection are highly susceptible to developing bacterial infection. Flu patients coinfected with bacteria have a high risk of serious illness and fatality (Bartley et al., 2022; Lee et al., 2022; Nolan et al., 2018; Bal et al., 2020). Coinfection with bacteria in hospitalized flu patients is recognized as the major determinant of mortality (Arranz-Herrero et al., 2023).

The respiratory tract possesses a comprehensive defense system against bacterial infection. Once inhaled bacteria enter the respiratory tract, they can be easily captured by mucus and cleared through the mechanical action of ciliated cells in the upper respiratory tract. Epithelial cells can secrete large amounts of surfactant proteins and antimicrobial peptides against bacterial infection at the same time. When the bacteria break through physical and chemical barriers, resident leukocytes, such as dendritic cells (DCs), alveolar macrophages (AMs), γδ T cells and invariant natural killer T (iNKT) response to invaded bacteria. These resident leukocytes can directly remove inhaled bacteria or secrete inflammatory mediators to recruit neutrophils and monocyte-derived macrophages to kill bacteria. Therefore, bacterial infection can be immediately eliminated by epithelial barriers and pulmonary immune cells during homeostasis (Neupane et al., 2020). But the first week of IFV infection can create a favorable pulmonary environment for secondary bacterial infections, thereby causing severe illness and high mortality (Neupane et al., 2020; Chen et al., 2021; Jia et al., 2018; Nickol et al., 2020; Langouët-Astrié et al., 2022). The poor outcome of viral-bacterial infection depends on numerous factors, including the damage of epithelial barriers, the impairment of antibacterial immune response (decreased phagocytosis, impaired ROS production, inhibition of activation, etc.) and the overactivated inflammatory response (Chen et al., 2021; Herrera et al., 2023; Martínez-Colón et al., 2019; Deinhardt-Emmer et al., 2020; Nickol et al., 2019; Gu et al., 2025). This review discusses the recent advances in our understanding of mechanisms that drive the pathogenesis of secondary bacterial infection following IFV infection and how this might inform future treatment options for preventing and treating viral-bacterial infection.

## 2 The epidemiology and bacterial spectrum of coinfection

Influenza epidemics occur annually and cause about 1 billion infections worldwide (WHO, 2019). More than 20% of influenza patients are complicated with bacterial pneumonia (Klein et al., 2016; Qiao et al., 2023). Seasonal influenza epidemics can lead to 3,200,000 cases of hospitalization globally each year (Paget et al., 2023). The incidence of coinfection in hospitalizations and ICU patients accounts for 17 and 28%, respectively (Qiao et al., 2023). The incidence of coinfection is higher in infants under 2 years of age and the elderly, especially in people over 70 years old. Patients coinfected with IFV and bacteria increase the mortality risk by 2.6 to 3.4 times compared with influenza single-infection (Arranz-Herrero et al., 2023; Qiao et al., 2023). More than 50% of the influenza-related deaths were attributed to bacterial infections during pandemics (Morens et al., 2008; Centers for Disease Control and Prevention (CDC), 2009; Estenssoro et al., 2010; Shieh et al., 2010). Approximately 23.8% influenza-associated deaths are attributed to bacterial infection in patients with seasonal IFV infection (Qiao et al., 2023; MacIntyre et al., 2018). *Streptococcus pneumoniae* (*S. pneumoniae*), *Staphylococccus aureus* (*S. aureus*), *Pseudomonas aeruginosa*, *Streptococcus pyogenes*, *Haemophilus influenzae*, *Klebsiella pneumoniae*, *Mycoplasma pneumoniae*, *Acinetobacter baumannii*, *Moraxella catarrhalis* and *Group A Streptococcus* are common bacteria during coinfection with IFV and bacteria (Arranz-Herrero et al., 2023; Klein et al., 2016; Shi et al., 2025). Gram-positive bacteria are the most frequent microorganisms identified in patients coinfected with IFV (Arranz-Herrero et al., 2023; Klein et al., 2016; Qiao et al., 2023; MacIntyre et al., 2018). *S. pneumoniae* is the most frequent pathogens followed by *S. aureus*. Both of them account for over 30% among all of bacteria (Arranz-Herrero et al., 2023).

## 3 Mechanisms of pathogenesis during post-influenza bacterial infection

Post-influenza virus infection is correlated with a deterioration of clinical outcome and higher mortality rates. Bacteria are easily eradicated and fail to cause serious injury during mild bacterial infection alone (Neupane et al., 2020). Initial influenza virus infection plays a crucial role in the pathogenesis of coinfection. The occurrence of antibacterial immunosuppression caused by initial influenza virus infection was considered the primary cause of coinfection. The severity of post-influenza bacterial infection is also associated with dysregulated immune response.

### 3.1 IFV infection creates a favorable environment for bacterial infection

#### 3.1.1 IFV infection disrupts the integrity of the epithelial barrier

The respiratory epithelium consists of multiple types of epithelial cells such as ciliated cells, secretory cells, basal cells, goblet cells and neuroendocrine cells, which cover the trachea and most of the proximal airways. It provides the first line of defense including physical barriers, secretory barriers and immune defense, against viral and bacterial infection. IFV infection causes multiple changes in the respiratory epithelial barrier, which weakens antibacterial defense and creates conditions for secondary bacterial infection that can be subverted by bacteria (LeMessurier et al., 2020). These changes can be divided into three major aspects, including damaging the integrity of respiratory barriers, suppressing antimicrobial immune responses and increasing bacterial colonization.

**Figure 1:**
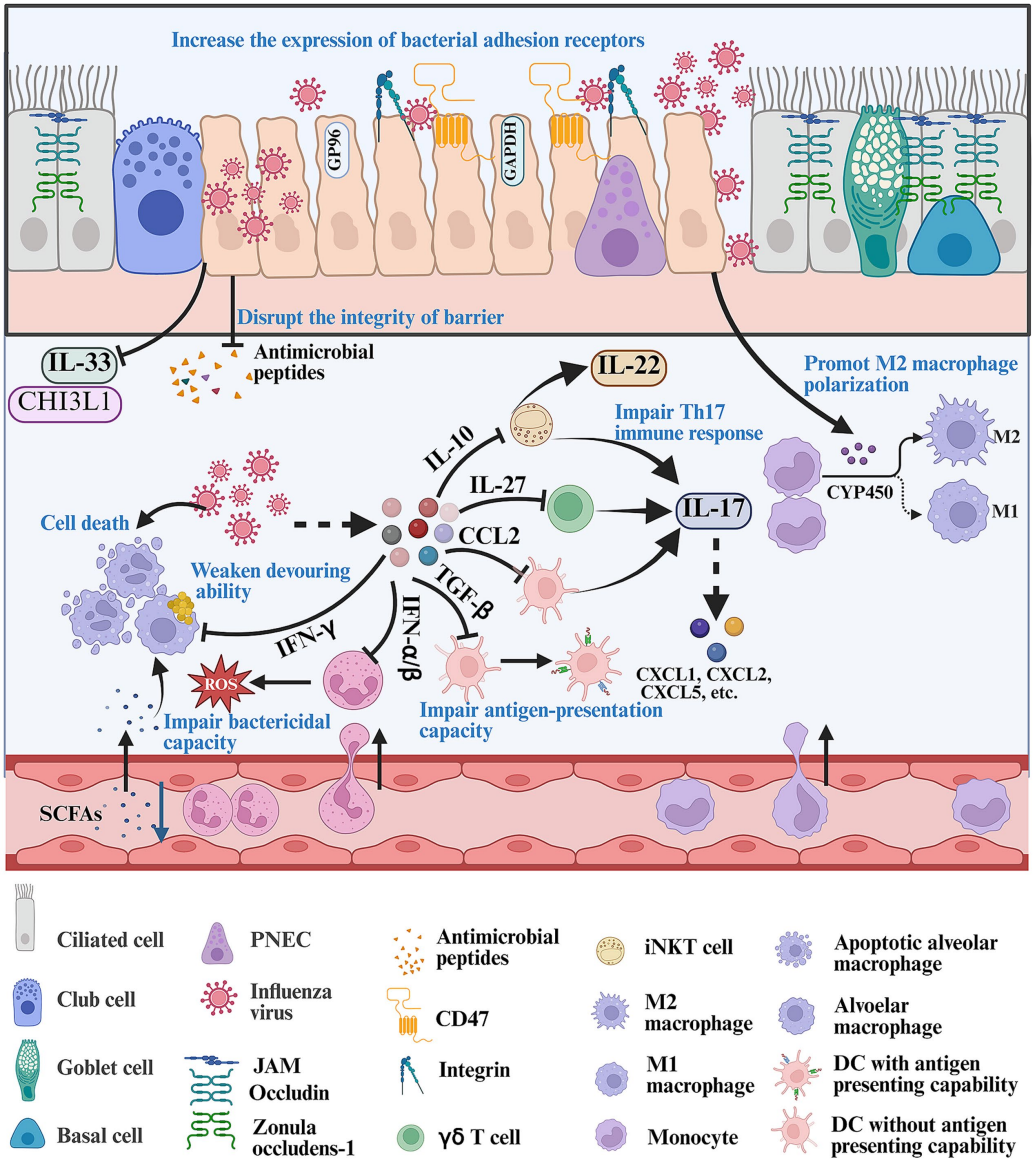
Mechanism about how IFV infection creates a favorable environment for bacterial infection. Firstly, IFV infection disrupts the integrity of respiratory barriers including reducing the expression of tight junction, IL-33, CHI3L1, etc., and decreasing the production of antimicrobial peptides. In addition, IFV infection can increase the expression of bacterial receptors such as CD47 and integrins, promoting bacterial infection. Once IFV breaks through the respiratory barrier, it can release various signals such as IL-10, IL-27, CCL2, TGF-β, IFN-α/β/γ and CYP450 metabolites, which impair the antibacterial ability of resident immune cells and recirculating innate immune cells. The figure was created via https://app.biorender.com/.

The respiratory mucosa serves as an initial barrier against invasive microorganisms and it can remove pathogens by mucin production and cilia activity (Chegini et al., 2023). The mucosa is constantly exposed to various bacteria. Invasive bacteria can be trapped and removed immediately by the secreted mucus. The physical barrier consists of multiple cell structures, including adherence junctions, gap junctions, tight junctions, desmosomes, and hemidesmosomes, which play an important role in defending against these invading pathogens (Gu et al., 2025). IFV infection can disrupt the integrity of barrier by reducing the expression of tight junction proteins and reorganizing zonula occludens-1 and occludin (Gu et al., 2025; Short et al., 2016; Golebiewski et al., 2011) (Figure 1). In addition, the epithelial cells can produce multiple antimicrobial peptides (AMPs) such as lipocalin 2, BPIFA1, LL-37, sPLA2-IIA, etc., which serve as a biochemical barrier to defense against bacterial penetration (Geitani et al., 2020). IFV infection can inhibit AMP production and facilitate secondary bacterial infection *in vivo*. Exogenous lipocalin 2 can assist in eliminating *S. aureus* in the lung during post-influenza *S. aureus* infection (Robinson et al., 2014; Lee et al., 2015). Chitinase-3-like 1 (CHI3L1) and IL-33 are protective factors associated with strengthening neutrophil-mediated immune response. IFV infection can inhibit the production of CHI3L1 and IL-33 in epithelial cells, thereby promoting secondary bacterial infection (Karwelat et al., 2020; Robinson et al., 2018b) (Figure 1).

Attachment of invasive bacteria to the respiratory tract is the first step during respiratory bacterial infection. Invasive bacteria utilize various surface proteins to bind to epithelial tissues. IFV infection can promote the expression of bacterial adhesion receptors. Integrins and CD47 are exploited as important receptors for bacterial infections. IFV infection can increase the expression of integrins and promotes group A *Streptococcus* (GAS) coinfection by inducing the expression of cyclophilin A or activating TGF-*β* signaling pathway (Li et al., 2015; Bai et al., 2022) (Figure 1). IFV infection can induce the expression of CD47 in nasal and bronchial epithelial cells in an NF-κB-dependent manner, with which gram-positive bacteria utilize fibronectin-binding protein to interact (Moon et al., 2024) (Figure 1). IFV infection can also increase the expression of fibronectin, platelet activating factor receptor (PAFr), intracellular adhesion molecule-1 (ICAM-1) and cell adhesion molecule 1 (CEACAM-1), which can serve as receptors for attaching to the host by nontypeable *Haemophilus influenzae* (NTHi), *S. aureus* and *S. pneumoniae* (Prystopiuk et al., 2018; Shukla et al., 2020; Novotny and Bakaletz, 2016; Wu et al., 2021; Vimalanathan et al., 2017). GP96 is a receptor for various bacterial pathogens such as *Escherichia coli*, *Listeria monocytogenes, S. pneumoniae* and *Neisseria gonorrhoeae*. IFV infection can induce the ectopic localization of GP96 in epithelial cells, which can be easily bound by AliA and AliB proteins of *S. pneumoniae* (Sumitomo et al., 2021). Another virulence factor, pneumococcal surface protein A (PspA), can interact with host-cell-derived GAPDH by PspA’s *α*-helical domain in dying cells to increase bacterial colonization during post-influenza *S. pneumoniae* infection (Park et al., 2021). IFV infection can destruct respiratory tract by recruiting inflammatory cells, which also provides binding sites for bacterial adhesion and promotes the proliferation of pathogens. IFV infection can recruit large numbers of Ly6C^hi^ inflammatory monocytes to the lung, which highly express tumor necrosis factor-related apoptosis-inducing ligand. Ly6C^hi^ inflammatory monocytes can induce apoptosis in epithelial cells, which causes damage to the lung barrier and increases bacterial colonization in the lung (Ellis et al., 2015). Post-influenza bacterial infection can induce the PINK1/Parkin-mediated mitophagy and facilitate the proliferation of IFV and *S. aureus* in pulmonary epithelial cells (Huo et al., 2025).

#### 3.1.2 IFV infection induces the dysfunction of lung-resident immune cells

There are various resident immune cells in the lung including unconventional T cells, resident innate immune cells and memory adaptive immune cells, which play an important role in defending against inhaled viruses, bacteria and other pathogens. Some resident pulmonary immune cells such as AMs, γδ T cells, iNKT cells and DCs, can respond to inhaled bacteria immediately and provide local protection against bacterial infection before recruitment of recirculating neutrophils and monocyte-derived macrophages (Braverman et al., 2022; Barker et al., 2021; Shenoy et al., 2020; Tavares et al., 2022). These resident immune cells can directly eliminate inhaled bacteria or affect multiple types of antibacterial immune responses by producing diverse cytokines such as IL-17, IFN-*γ*, IL-22, etc. IFV infection can suppress their antibacterial capacity and alter their response to bacterial infection.

AMs are resident pulmonary macrophages and they are the main immune cells present in the alveoli during homeostasis (Neupane et al., 2020). They patrol the alveoli, clean the alveolar spaces and crawl to kill inhaled bacteria to maintain homeostasis, thereby preventing severe bacterial infection during homeostasis (Neupane et al., 2020; Tam et al., 2020). But IFV infection compromises the antibacterial ability of AMs including impairing the ability to crawl, decreasing the expression of phagocytosis receptor, weakening devouring ability and inducing cell death of AM, thus enabling noninvasive bacteria to cause fatal pneumonia in influenza (Verma et al., 2020; Sencio et al., 2020). NK, NKT and T cells can rapidly secrete IFN-*γ* during IFV infection. IFN-γ can impair the function of AM including impairing the ability to crawl and inhibiting the expression of phagocytic receptor during post-influenza bacterial infection (Casanova et al., 2024; Schmolke et al., 2021) (Figure 1). The class A scavenger receptor macrophage receptor with collagenous structure (MARCO) is an important scavenger receptor that recognizes and binds gram-positive and gram-negative bacteria, and is the main receptor for phagocytosis of particles and exogenous bacteria by macrophages. Blocking IFN-*γ* signaling can promote the expression of MARCO by inhibiting Akt activation and restore the ability of AM migration (Neupane et al., 2020; Wu et al., 2017). IL-6 also promotes macrophage phagocytosis by increasing the expression of MARCO during post-influenza *S. pneumoniae* infection (Gou et al., 2019). Microbiota-derived metabolites such as rhamnose, indole 3-propionic acid and short chain fatty acids (SCFAs) are recognized as important players promoting macrophage phagocytosis and protecting against bacterial infection (Sencio et al., 2020; Li et al., 2024; Huang et al., 2022; Schuijt et al., 2016). SCFAs are known to promote phagocytosis of AMs by interacting with the GPR43 receptor, and protect mice from bacterial infection during *S. pneumoniae* or *Klebsiella pneumoniae* infection (Schuijt et al., 2016; Le Guern et al., 2023; Machado et al., 2022; Galvão et al., 2018). Gut dysbiosis and related metabolic dysfunction, especially reduction in SCFAs, can increase susceptibility to secondary bacterial infection following IFV infection (Chen Q. et al., 2025) (Figure 1). *Blautia faecis* DSM33383 can produce large amounts of acetic acid and the intragastrical administration of *Blautia faecis* DSM33383 can protect mice from post-influenza bacterial infection (Verstraeten et al., 2022). In addition, IFV infection can induce cell death of resident AMs and the majority of AMs would be lost in a week (Ghoneim et al., 2013). GM-CSF is an important factor promoting the maturation, differentiation and activation of AMs (Chen Y. et al., 2023). GM-CSF treatment increases the number of AMs and promotes bacterial clearance in the lung during post-influenza *S. aureus* infection (Ghoneim et al., 2013). Deficiency in GM-CSF’s receptor (*Csf2rb*^−/−^) abrogates bacterial clearance during post-influenza *S. pneumoniae* infection (Verma et al., 2020). IL-1 signaling can also contribute to the maintenance of the number of AMs and promote bacterial clearance in the lung (Bansal et al., 2018).

Lung-resident γδ T cells account for 8–20% of resident pulmonary lymphocytes and aid in eliminating bacteria (Min and Shilian, 2017; Cheng et al., 2012). Mice are susceptible to bacterial infections and develop deadly pneumonia after IFV infection in a week (Herrera et al., 2023; Yi et al., 2022). γδ T cells are a major source of IL-17A within these time points, because Th17 immune response develops slowly (Cao et al., 2014). IL-17A can enhance antimicrobial capacity by contributing to MIP-2-driven neutrophil recruitment, anti-microbial peptide secretion and enhancement of the mucosal barrier function (Mills, 2023). IFV infection induces the secretion of IL-27 that inhibits the *Streptococcus*-induced IL-17A expression via suppressing the activation of STAT1 signaling pathway in *γ*δ T cells and promotes the development of secondary pneumococcal pneumonia (Cao et al., 2014; Robinson et al., 2015; Lee et al., 2017) (Figure 1). But dysregulated, chronic IL-17 signaling promotes excessive inflammation in lungs by sustaining neutrophil infiltration and triggering other pro-inflammatory pathways.

Invariant natural killer T (iNKT) cells are tissue-resident lymphocytes and account for approximately 5% of lymphocytes in mouse lung, which can response to *S. pneumoniae* infection and secrete IFN-*γ* and IL-17A within 13 h (Crosby and Kronenberg, 2018). IFN-*γ* is involved in activating NK cells that can assist in protecting against secondary bacterial infection following IFV infection (Casanova et al., 2024; Small et al., 2010). IFV infection dampens the activation of iNKT cells by inducing the production of IL-10, accompanied by a decrease in the production of IFN-γ and IL-17A (Barthelemy et al., 2017) (Figure 1). The activation of iNKT cells can prevent pneumococcal outgrowth during post-influenza bacterial infection by increasing the production of IFN-γ and IL-17A (Barthelemy et al., 2016). Thus, IFN-γ acts as a double-edged sword: it is a vital activating signal for mobilizing NK cells against post-influenza bacterial infection, yet it can simultaneously exacerbate the immunosuppressive state by further inhibiting the antibacterial functions of AMs, thereby worsening the outcome of influenza-bacterial coinfection. Additionally, iNKT cells can secrete IL-22 that is beneficial in limiting lung inflammation and alleviating ALI during post-influenza bacterial infection (Paget et al., 2012; Ivanov et al., 2013).

There are three types of resident DCs in the lung during homeostasis, including conventional DC1 (cDC1), conventional DC2 (cDC2) and pDC. DCs exhibit antigen-presentation capacity and secrete immunogenic cytokines such as IL-17 and IFN-*γ*, which strengthen both innate and adaptive immunity to promote bacterial clearance (Ardain et al., 2020). Prior IFV infection can impair its antigen-presentation capacity, affect self-renewal and influence the activation of antimicrobial immune responses. DCs can produce TGF-*β* and induce the accumulation of Treg cells during a primary IFV infection, which induces the differentiation of DCs into paralyzed DCs. These paralyzed DCs exhibit high amounts of Blimp1 that can induce tolerogenic functions in DCs, and low levels of IRF4 that can promote antigen presentation to CD4^+^ T cells. For these paralyzed DCs, MHC II-mediated T cell priming is defective for at least three weeks and antigen-presentation capacity is defective *in vitro* (Roquilly et al., 2017) (Figure 1). DCs must be constantly replenished by newly recruited cells from the bone marrow. Fms-like tyrosine kinase 3 ligand (Flt3-L) is a critical cDC differentiation factor. IFV infection can decrease the production of Flt3-L in the bone marrow and blood, which results in lower generation of cDC progenitors in the bone marrow, accompanied by a decrease of cDCs (cDC1 and cDC2) in the lung. Overexpression of Flt3-L promotes the cDC progenitors’ production in the bone marrow, replenishes cDCs in the lung and protects against post-influenza pneumococcal infection (Beshara et al., 2018). CC chemokine receptor 2 (CCR2) is an important receptor recruiting DCs and macrophages by interacting with CCL2. IFV infection can secrete high levels of CCL2. CCR2^−/−^ mice exhibit decreased accumulation of cDC2 and increased accumulation of cDC1, accompanied by increased release of IL-17 in the lung during post-influenza *S. aureus* infection. Antagonizing IL-17 partially abrogated the protection seen in CCR2^−/−^ mice (Gurczynski et al., 2019) (Figure 1).

#### 3.1.3 IFV infection impairs the antimicrobial capacity of recirculating innate immune cells

Recirculating neutrophils and monocyte-derived macrophages are primary innate immune cells against bacterial infection. They can respond to inhaled bacteria and be recruited to the lung immediately. Inhaled bacteria can be attacked and eliminated by recirculating neutrophils and monocyte-derived macrophages with their weapons such as ROS, proteases, etc. IFV infection can alter their response to inhaled bacteria and their ability to kill bacteria (Martínez-Colón et al., 2019; Ho et al., 2018; Berg et al., 2017).

Neutrophils, one of the most numerous circulating leukocytes, are required for eliminating bacteria in BALB/c mice coinfected with IFV and *S. pneumoniae* (Palani et al., 2023). They devour bacteria and kill them by producing high levels of ROS. A clinical study indicates that neutrophils isolated from airway fluid of patients coinfected with influenza and bacteria exhibit high levels of activation markers (HNE and MPO), but their ability to kill *H. influenzae* and *S. aureus* is dampened *in vitro* (Grunwell et al., 2019). Neutrophils isolated from mice also exhibit low levels of ROS and neutrophil elastase during post-influenza bacterial infection (Jie et al., 2023; Ishikawa et al., 2016; Sun and Metzger, 2014). Furthermore, IFV infection impairs their ability to devour and kill invasive bacteria during post-influenza *P. aeruginosa* infection *in vivo* (Jie et al., 2023). Type I IFN signaling can weaken the capacity of neutrophils to kill *S. aureus* during post-influenza *S. aureus* infection on day 7 after IFV infection (Shepardson et al., 2016) (Figure 1).

Recirculating macrophages are divided into proinflammatory M1 macrophages characterized by high levels of ROS and NO production, and immunosuppressive M2 macrophages (Chen S. et al., 2023). M1 macrophages play a vital role in resisting bacterial invasion. CYP450 metabolites are ligands for peroxisome proliferator-activated receptor *α* (PPARα). IFV infection induces the production of CYP450 metabolites (5,6-diHETrE, 8,9-diHETrE, 11,12-diHETrE and 14,15-diHETrE) and triggers the activation of the PPARα signaling pathway, which promotes polarization of M2 macrophages and dampens bacterial clearance during post-influenza *S. aureus* infection (Tam et al., 2020; Lucarelli et al., 2022) (Figure 1). STAT2 signaling is also linked to inhibiting macrophage (M1 and M2) accumulation and impairing bacterial clearance during post-influenza bacterial infection. Neutralizing IFN-*γ* (M1) and/or Arginase 1 (M2) can reduce bacterial clearance in Stat2^−/−^ mice during post-influenza bacterial infection (Gopal et al., 2018). SHP2-deficient macrophages exhibit enhanced polarization towards an M2 phenotype and a decreased antibacterial capacity during post-influenza *S. aureus* infection (Ouyang et al., 2019).

### 3.2 Dual infection causes severe immunopathological damage

#### 3.2.1 The overactivation of the inflammatory response

The immune system is in a hyperactive state during post-influenza bacterial infection, which can be activated by recognizing the components of IFV following bacteria. The overactivation of the inflammatory response is a key pathophysiological factor causing acute lung injury (ALI) and a high mortality rate (Klemm et al., 2017; Damjanovic et al., 2013; Jia et al., 2023). Post-influenza bacterial infection occurs in the early stage of IFV infection and causes the mortality of mice within days (Jia et al., 2018; Herrera et al., 2023; Li-Juan et al., 2022). The innate immune system plays a more important role in driving the development of post-influenza bacterial infection during the early stage of infection. This study mainly discusses the overactivation of inflammatory response during coinfection with IFV and bacteria.

**Figure 2:**
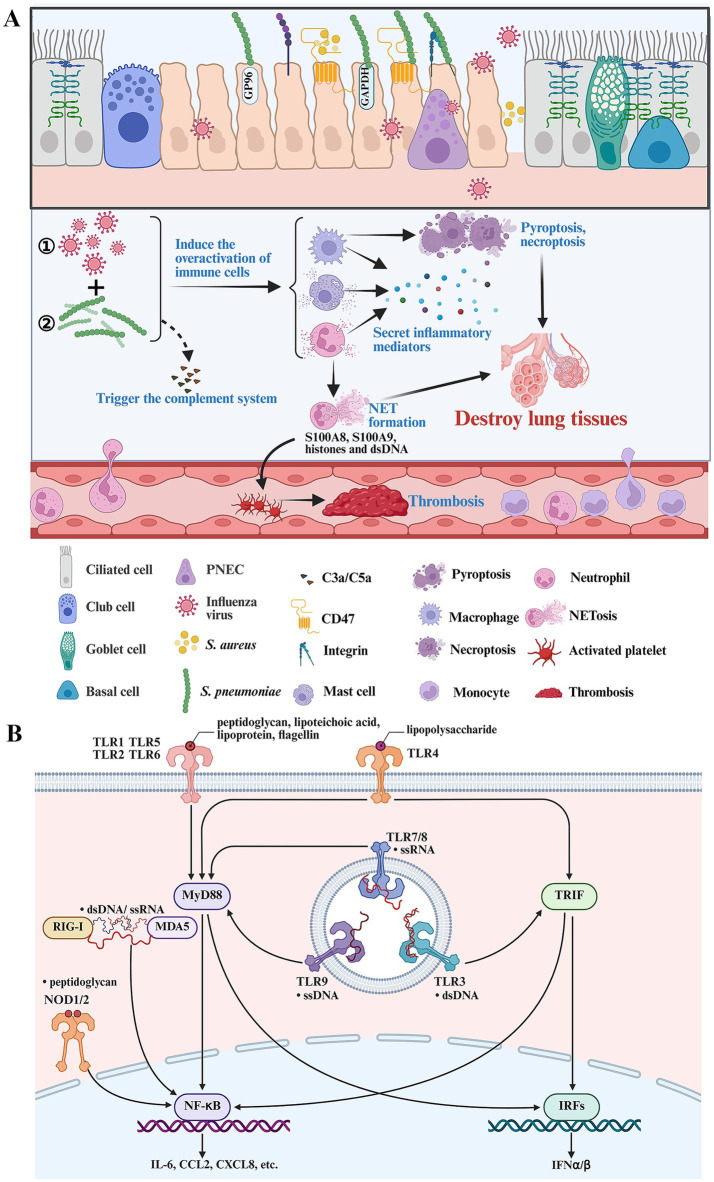
Key immune cells and signaling pathways in driving disease progression during secondary bacterial infection following IFV infection. **(A)** Post-influenza bacterial infection activates the complement system and immune cells. Activated macrophages, neutrophils and mast cells play an important role in driving the formation of overactivated inflammatory response during post-influenza bacterial infection. Activated neutrophils lead to NET formation and immune thrombosis by releasing S100A8, S100A9 and histones. Postinfluenza bacterial infection can result in pyroptosis and necroptosis. **(B)** The components of IFV and bacteria can be recognized by various PRRs and induce overactivated inflammatory response by activating multiple inflammatory signaling pathways. The figure was created via https://app.biorender.com/.

Different complement factors can bind to the surface of IFV or bacteria to trigger the complement system. During the early stage of infection, the components of IFV and bacteria can induce the release of C3a and C5a by activating the lectin pathway and the alternative pathway (Santos et al., 2021; Syed et al., 2020). C3a and C5a are potent chemoattractants recruiting neutrophils, macrophages, etc., to infectious sites. In addition, C3a and C5a can activate neutrophils, mast cells and basophils to release histamine, leukotriene, ROS, etc., increasing vascular permeability and destroying the epithelial-endothelial barrier (Figure 2A). Post-influenza bacterial infection can induce an overactivated inflammatory response by activating the complement system. The overactivation of complement is associated with ALI and high mortality in mice coinfected with IFV and *S. aureus* (Jia et al., 2023).

The components of IFV and bacteria can be sensed by pattern recognition receptors (PRRs), including Toll-like receptors (TLRs), nucleotide binding oligomerization domain (NOD)-like receptors (NLRs), retinoic acid-inducible gene (RIG)-I-like receptors (RLRs) or DNA-sensing molecules. Post-influenza bacterial infection induces a dual inflammatory response in epithelial or immune cells (neutrophils, macrophages, etc.) by sensing the components of IFV and bacteria, respectively (Yuki and Koutsogiannaki, 2021; Verma et al., 2022). Viral single-stranded RNA (ssRNA) can be sensed by RIG-I, MDA5, TLR7 and TLR8 (Figure 2B). In addition, IFV can produce double-stranded RNA (dsRNA) during viral replication, which can be sensed by RIG-I, MDA5 and TLR3 (Figure 2B). The components of gram-positive bacteria such as peptidoglycan, lipoteichoic acid, lipoproteins and bacterial DNA, can be sensed by TLR2, NOD1/NOD2 and TLR9 (Figure 2B). The components of gram-negative bacteria such as peptidoglycan, lipopolysaccharide, flagellin and bacterial DNA, can be sensed by TLR2, NOD1/NOD2, TLR4, TLR5 and TLR9 (Figure 2B). Therefore, post-influenza bacterial infection induces a more aggressive inflammatory response by activating more inflammatory signaling pathways compared with influenza or bacterial infection alone (Jie et al., 2023; Chen B. et al., 2025). A recent longitudinal transcriptional study indicated that the top upregulated differentially expressed genes during post-influenza *S. pneumonia* infection are involved in inflammatory response (Cohn et al., 2024). Neutrophils and macrophages are the predominant drivers of overactivated inflammatory response during severe bacterial lung infection (Xiao et al., 2025). Recently, a single-cell RNA sequence study indicated that neutrophils, interstitial macrophages and classical monocytes are key drivers of cytokine storm during post-influenza *S. aureus* infection (Lei et al., 2025). But the comprehensive immune response within bronchoalveolar lavage fluid (BALF) samples from flu patients with bacterial pneumonia and healthy controls needs to be verified using single-cell RNA sequencing technology in the future.

Neutrophils play a crucial role in clearing bacteria during the early stages of bacterial invasion following influenza infection. The capability of neutrophils to eliminate bacteria can be impaired by initial influenza virus infection. However, these neutrophils still possess pro-inflammatory properties. In addition, both initial influenza virus infection and secondary bacterial infection recruit large numbers of neutrophils to the lungs (Lei et al., 2025). Therefore, neutrophils inevitably cause tissue damage during post-influenza bacterial infection. Neutrophils can directly destroy normal lung tissues and release damage-associated molecular patterns (DAMPs) to exacerbate inflammatory response (Lei et al., 2025; Burn et al., 2021). Oxidative burst can induce the formation of neutrophil extracellular traps (NETs) accompanied by releasing high levels of ROS, histones and proteases, which contribute to lung damage. NET formation in mice coinfected with IFV and bacteria contributes to endothelial injury and lung damage (Yi et al., 2022; Narayana Moorthy et al., 2013). Activated neutrophils and NET formation can also release high levels of proinflammatory mediators including S100A8, S100A9, histones and dsDNA, which can induce overactivated inflammatory responses by activating TLR4, TLR2 or cGAS-STING signaling pathways (Xiao et al., 2025; Pruenster et al., 2016; Ramasubramanian et al., 2022; Huang et al., 2013; Wilson et al., 2022; Katsoulis et al., 2024). Patients coinfected with IFV and bacteria increase the risk of coagulation disorders (Brown et al., 2024). Neutrophil activation is associated with widespread pulmonary thrombosis and alveolar oedema during post-influenza *S. pneumonia* infection (Walters et al., 2015). The release of S100A8/A9 and histones in neutrophils binds to GPIb*α* or TLR2/4 receptors of platelets, which subsequently drive the formation of immune thrombosis (Colicchia et al., 2022; Semeraro et al., 2011) (Figure 2A). Most of recirculating inflammatory monocytes are recruited into the lung and differentiate into macrophages during infection. Activated macrophages can generate reactive nitrogen species (RNS), TNF-α, etc., to augment lung damage and exacerbate disease progression. Activated macrophages can also produce matrix metalloproteinases such as MMP-9 and MMP-12, to damage the alveoli during post-influenza bacterial infection (Rojas-Quintero et al., 2018; Villeret et al., 2020; Lee et al., 2018).

Mast cells are an important part of the mucosal immune system in the lung and can secrete high levels of cytokines and chemokines, responding to IFV and bacterial infection (Hu et al., 2012; Malaviya et al., 1996). Post-influenza *S. aureus* infection can inhibit autophagy and facilitate the secretion of inflammatory mediators in mast cells by activating the PI3K/Akt signaling pathway (Tang et al., 2023) (Figure 2A). Suppressing the PI3K/Akt signaling pathway can inhibit the production of inflammatory mediators and alleviate ALI caused by secondary *S. pneumoniae* or *S. aureus* infection following IFV infection (Tang et al., 2023; Yang et al., 2019).

#### 3.2.2 Pyroptosis and necroptosis

Pyroptosis is an immunogenic form of cell death and can assist in eliminating pathogens. But it can also drive inflammatory damage by releasing inflammatory mediators (Corry et al., 2022; Zheng et al., 2023) (Figure 2A). Pyroptosis is characterized by inflammasome and caspase activation. Inflammasome activation including NLRP3/ASC, Pyrin/ASC, AIM2/ASC, NLRC4 and NLRP1, can contribute to caspase-1 activation that cleaves gasdermin D (GSDMD), exposes the N domain of GSDMD and results in pore formation (Zhao et al., 2011; Bai et al., 2025). In addition to classic inflammasome-dependent pyroptosis, caspases-4/5/8/11 can also directly cleave GSDMD through non-canonical pathways and initiate pyroptosis (Shi et al., 2015; Zanoni et al., 2016; Broz et al., 2019). TLR2-MYD88-NLRP3 axis mediates IL-1β production during post-influenza *S. pneumoniae* infection (Rodriguez et al., 2019). NLRP3 activation can increase bacterial burden and inflammatory response, which is associated with poor outcome in mice coinfected with IFV and bacteria (Shi et al., 2020). While ASC activation results in increased inflammation and mortality, but contributes to bacteria clearance during post-influenza *S. aureus* infection (Robinson et al., 2018a). The E3 ubiquitin ligase NEDD4 can promote GSDMD-mediated pyroptosis and result in poor outcome during post-influenza *S. pneumoniae* infection (You et al., 2023). IL-4 exerts protective effects against post-influenza *S. pneumoniae* infection by suppressing GSDMD-induced pyroptosis (Peng et al., 2021).

Necroptosis is a form of inflammatory cell death that aggregates tissue damage by releasing DAMPs and amplifying inflammation (Pasparakis and Vandenabeele, 2015) (Figure 2A). Necroptosis is initiated by death receptors (e.g., TNFR1) or pathogen sensors (e.g., ZBP1), which subsequently triggers RIPK1-RIPK3-MLKL cascade. MLKL forms pore-like structures, therefore disrupting membrane integrity and resulting in osmotic cell lysis, accompanied by the release of DAMPs (Yuan and Ofengeim, 2023). Pore-forming toxins (PFTs), such as pneumolysin produced by *S. pneumoniae*, are known factors to induce necroptosis of lung epithelial cells (LECs) and are required for inducing necroptosis during post-influenza *S. pneumoniae* infection. IFV infection causes residual oxidative stress that enhances susceptibility to bacterial-toxin-mediated necroptosis (Gonzalez-Juarbe et al., 2020). IFV infection can also potentiate *S. pneumoniae* infiltration in the heart, and induce oxidative stress to enhance bacterial toxin-induced necrotic cell death and cause proteomic remodeling of the heart (Platt et al., 2022). In addition, IFV infection can activate PPARα signaling, mediate RIPK3-dependent necroptosis and result in increased mortality during post-influenza *S. aureus* infection (Tam et al., 2020). Large quantities of Z-RNAs, type I interferon and dsDNA are produced during post-influenza bacterial infection. All of these factors can induce ZBP1-mediated necroptosis. Whether ZBP1-mediated necroptosis can result in increased mortality during post-influenza bacterial infection remains unknown.

## 4 Treatment options

As our understanding of the molecular mechanisms underlying virus-bacteria coinfection deepens, these discoveries present opportunities for developing novel therapeutic approaches and preventive strategies. Significant progress has been made in multiple control strategies targeting either pathogens or hosts.

### 4.1 Vaccination

Influenza vaccination is the most effective strategy for preventing IFV infections (Minozzi et al., 2022). Vaccines against IFV can effectively reduce influenza-associated secondary bacterial infections as well. Live influenza vaccine can protect mice from post-influenza *S. pneumoniae* infection (Desheva et al., 2022). However, live influenza vaccine could increase the risk of bacterial colonization. Multiple clinical studies indicate that live influenza vaccines can increase nasopharyngeal pneumococcal carriage and density (Peno et al., 2021; Peno et al., 2025; Glennie et al., 2016). Live attenuated influenza vaccines can also enhance colonization of *S. pneumoniae* and *S. aureus* in mice (Mina et al., 2014). Vaccines against IFV can impair antibacterial immune response, which is similar to prior IFV infection (Jochems et al., 2018).

Vaccination with the antigen of *S. pneumoniae* or *P. aeruginosa* such as PspA or PcrV protein can also exert protective effects against post-influenza bacterial infection in animal models (Majumder et al., 2024; Wu et al., 2023). Vaccination with PspA protein can significantly increase the number of AMs and promote bacterial clearance in the lung (Majumder et al., 2024). Prior *S. pneumoniae* infection can protect against different serotypes of *S. pneumoniae* infection following IFV infection by inducing a cross-reactive Th17 response (Li et al., 2023). Vaccination with a conserved NTHi antigen, protein 0529, can protect mice from post-influenza NTHi infection by increasing Th17 response (Zhang et al., 2023). In addition, virus-bacterial vaccines can also provide protection against post-influenza bacterial infection in a mouse model (Desheva et al., 2022; Li et al., 2023). Whole-inactivated influenza A and pneumococcal vaccines can increase IFV-specific CD8^+^ T cell response in the lung (David et al., 2019). These bacterial vaccines can effectively activate an antibacterial immune response. However, most of these vaccines target single bacterial infections and are not broad-spectrum.

### 4.2 Antivirals and antibiotics

Antivirals represent an essential strategy for the prevention and treatment of influenza. Antivirals can decrease influenza-associated morbidity, complications and mortality (Arnold et al., 2025; Uyeki et al., 2019). Preceding IFV infection in the lung is a major risk factor for secondary bacterial infection. It destroys the respiratory barriers, increases bacterial adhesion and suppresses antibacterial immune response facilitating bacterial infection to occur. Timely antiviral treatment can effectively prevent secondary bacterial infections and also benefits severe influenza complicated with bacterial infection (Uyeki et al., 2019; Wang et al., 2023). Antivirals, such as peramivir and oseltamivir, can reduce the incidence of secondary bacterial infection, mitigate virus-induced injury and protect mice from post-influenza bacterial infection (Lei et al., 2025; Zhao et al., 2021; Onishi et al., 2015). At present, more and more new antivirals such as suraxavir marboxil and onradivir, are available for the treatment of IFV infection (Wang et al., 2025; Yang et al., 2024). Additionally, neutralizing HA antibodies can also alleviate ALI and increase the survival rate of mice during post-influenza bacterial infection (van Someren Gréve et al., 2018; Robinson et al., 2019). Antivirals can decrease viral load and alleviate virus-induced injury, thus reducing the incidence of secondary bacterial infection. Therefore, timely antiviral treatment is needed to control viral replication.

Secondary bacterial infection can amplify overactivated inflammatory response and further ruin the normal lung tissues. The timely antibiotic treatment against susceptible bacteria benefits flu patients with bacterial infection (Wang et al., 2023). Antibiotic treatment alone can reduce bacterial load and alleviate lung damage caused by post-influenza bacterial infection (Verma et al., 2019; Song et al., 2022). But a study indicates that penicillin G treatment alone cannot increase the survival rate of mice during post-influenza *s. aureus* infection (Song et al., 2022). Antibiotic treatment can effectively inhibit bacterial load, but it is insufficient to control the replication of IFV and the overactivated inflammatory response.

### 4.3 Anti-adhesion

Anti-adhesion therapy can prevent microbial adhesion to cells or tissues, and has become a promising strategy in infectious diseases (Asadi et al., 2019). IFV infection can upregulate the expression of multiple adhesion receptors, promoting bacterial adherence. Inhibiting the expression of adhesion receptors can effectively disrupt bacterial adherence (Vimalanathan et al., 2017; Ishikawa et al., 2022). WEB-2086, an antagonist of PAFr, can decrease the adhesion of *S. pneumoniae* and NTHi to cigarette smoke extract-treated bronchial epithelial cells (Shukla et al., 2016). CV-3988 is also a specific antagonist targeting PAFr. It can inhibit mild steel welding fumes-mediated pneumococcal adhesion *in vitro* and *in vivo*. Some traditional Chinese medicines such as Liu Shen Wan and Lianhuaqingwen can reduce *S. aureus* adherence to IFV-infected respiratory epithelial cells by downregulating the expression of CEACAM1, ICAM-1 and integrin-α5, therefore protecting mice from post-influenza *S. aureus* infection (Zhao et al., 2021; Song et al., 2022; Du et al., 2021). Targeting adhesion receptors may be a new potential therapy in preventing and treating influenza bacterial coinfection. However, bacteria utilize multiple receptors to infect hosts, rendering single anti-adhesion therapies ineffective. In addition, anti-adhesion agents must be administered during the early stages of infection or preventatively. In clinical practice, this therapeutic window is frequently missed due to delayed presentation.

### 4.4 Neutralizing antibodies against proinflammatory cytokines

Uncontrolled pathogens can induce the overactivation of inflammatory response and contribute to severe organ injury. In addition to controlling the replication of pathogens, it’s important to inhibit the overactivated inflammatory response during post-influenza bacterial infection (Damjanovic et al., 2013; Tavares et al., 2017). Anti-IL-6 and anti-IL-1 antibodies have been widely used for the treatment of COVID-19 (Batista and Foti, 2021). Some neutralizing antibodies such as tocilizumab and anakinra, can inhibit systemic inflammation and decrease the risk of invasive mechanical ventilation or death in patients with COVID-19 (Guaraldi et al., 2020; Kyriakoulis et al., 2021). Single IFN-*γ* neutralization can reduce local bacterial load in the lung. Concomitant neutralization of IFN-γ and IL-6 can inhibit bacterial load, reduce the secretion of cytokines and alleviate the degree of pneumonia as well as bacteremia during post-influenza *S. pneumoniae* infection (Sharma-Chawla et al., 2019). The use of such antibodies carries a risk of immunosuppression, potentially weakening the body’s ability to clear bacteria and increasing the risk of uncontrolled infection. Furthermore, the heterogeneity of patient immune responses complicates distinguishing individuals who may benefit from cytokine blockade versus those who may be harmed by it, thereby complicating clinical trial design and patient stratification.

### 4.5 Other host-directed therapies

Therapies that modulate the overactivated inflammatory response, can prevent the emergence of severe immunopathology in infectious disease. Anti-C5aR treatment can significantly increase the survival rates of mice and mitigate lung injury by reducing the release of inflammatory mediators such as IFN-γ, TNF-*α*, IL-6 and IL-8 (Jia et al., 2023). Inhibition the activation of NLRP3 by MCC950 can decrease the secretion of G-CSF, MIP-1α, KC and IL-1β during post-influenza *S. aureus* infection. However, MCC950 cannot increase the survival rate of mice (Robinson et al., 2018a). DNase I can mitigate lung injury by reducing the expression of MCP-1, IL-1β and ICAM-1 in coinfected animals (Yi et al., 2022).

### 4.6 Traditional Chinese medicines

Traditional Chinese medicines have been recommended for the treatment of IFV infection since 2009. A Chinese formula contains various active ingredients and plays a versatile role in the treatment of IFV infection. Some of them possess antiviral, antibacterial and anti-inflammatory effects (Zhao et al., 2023; Chen and Wang, 2023; Yang et al., 2024; Zhang J. et al., 2025; Zhang B. et al., 2025). Some Chinese formulas such as Liu Shen Wan, Lianhuaqingwen capsule and Jing-Yin-Gu-Biao formula, have been found to protect mice from post-influenza bacterial infection (Lei et al., 2025; Zhao et al., 2021; Song et al., 2022; Du et al., 2021). Liu Shen Wan and Lianhuaqingwen capsule can inhibit replication of IFV, virus-induced overactivated inflammatory response and the expression of adhesion receptor (Zhao et al., 2021; Du et al., 2021; Ma et al., 2020; Yang et al., 2020). Jing-Yin-Gu-Biao formula can suppress the overactivation of neutrophils and NETosis during post-influenza *S. aureus* infection (Lei et al., 2025). A homogeneous polysaccharide from *Houttuynia cordata* can protect mice from post-influenza *S. aureus* infection, which can reduce excessive intestinal complement activation (C3a and C5a) and block the NLRP3 pathway, helping regulate the Treg/Th17 cell balance in the gut-lung axis (Li et al., 2025). Traditional Chinese medicines are also promising optional agents for the treatment of post-influenza bacterial infection.

### 4.7 Combination therapy

It is uncertain whether the use of antiviral drugs alone can reduce mortality in patients with severe influenza due to the lack of data from clinical trials (Gao et al., 2024). Multiple combination strategies such as antivirals + antivirals, antivirals + monoclonal antibodies, antivirals + anti-inflammatory agents, antivirals + antibiotics, have been considered for the treatment of severe influenza (Koszalka et al., 2022; Zhou et al., 2025; Lim et al., 2020; Lee et al., 2021). Severe influenza especially coinfected with IFV and bacteria is caused by multiple pathogenic factors and should be considered combination therapy. The combination of antibiotics and oseltamivir can inhibit inflammatory response, shorten the length of hospital stay and decrease the incidence of ICU admission and secondary bacterial infections (Ishaqui et al., 2021; Ishaqui et al., 2020; Lee et al., 2017). Clarithromycin-Naproxen-Oseltamivir combination can also decrease the mortality and length of hospitalization in hospitalized patients with IFV infection (Lee et al., 2021; Hung et al., 2017). The combination of antibiotics and clindamycin can increase the survival rate of mice during post-influenza *S. pneumoniae* infection (Li et al., 2018). Combined oseltamivir and traditional Chinese medicine are superior to oseltamivir treatment alone in inhibiting overactivated inflammatory response and alleviating ALI during post-influenza bacterial infection (Lei et al., 2025). Combination therapy may be the most important strategy for the treatment of coinfection with IFV and bacteria.

## 5 Future directions

This review summarizes epidemiology, pathogenesis and therapeutic strategies during post-influenza bacterial infection. To advance this field, future research must address several critical topics. First, several mechanisms that may play an important role in the co-pathogenesis of influenza-bacterial infections were summarized in this study. Our findings are constrained by the scarcity of large-scale human transcriptomic datasets. A crucial next step is the generation of transcriptomic data from diverse cohorts to enhance the generalizability of findings from the preclinical study. Second, clinical studies have demonstrated that combination therapy holds significant potential for treating severe influenza. Rational combination therapies concurrently addressing pathogen clearance and host immunomodulation show superior potential for managing coinfection complexity. The promising benefits of combination therapies in influenza-bacterial infection have not been rigorously tested in clinical settings. Large-scale, multicenter clinical trials are required to ultimately evaluate the safety and synergistic potential of combination therapies.

## 6 Conclusion

IFV infection severely threatens global health, with secondary bacterial coinfection dramatically amplifying morbidity and mortality through synergistic pathogenesis. Crucially, IFV establishes a permissive environment for bacterial invasion by disrupting respiratory epithelial integrity and suppressing innate antimicrobial immunity, underscoring the imperative for prompting viral control to prevent secondary complications. Once coinfection ensues, dysregulated host-pathogen interactions trigger hyperinflammation wherein recruited neutrophils and macrophages paradoxically become key drivers of immunopathology, which fuel tissue damage despite their defensive roles. This mechanistic insight necessitates therapeutic strategies beyond conventional antimicrobials: modulating pathological immune responses, particularly targeting the neutrophil-macrophage axis and associated hyperinflammatory cascades, represents a promising host-directed approach to mitigate organ damage.
